# Keyword Index for Volume 92

**DOI:** 10.1038/sj.bjc.6602670

**Published:** 2005-06-14

Keyword Index for Volume 92

11*β*HSD-1/2 1927

11p15.5 2216

17*β*-hydroxysteroid dehydrogenase 547

^18^F-FDG PET/CT 1046

1p 1553

5-aza-2′-deoxycytidine 2024

5-fluorouracil 24, 259, 634, 722, 832, 1644, 2122

5-FU 1850

80 kDa fragment 2018

acitretin 696

activation signaling 305

acute renal failure 480

acute-phase protein response 673

ADCC 342

adenoma 736

adenovirus 882

adhesion 729

adjuvant 24, 843

adjuvant chemotherapy 259, 2107

adjuvant radiochemotherapy 1051

adjuvant therapy 467, 1655

adrenal incidentalomas 1104

advanced biliary cancer 1650

advanced breast cancer 639

advanced gastric cancer 246, 1644

advanced ovarian cancer 1019

Africa 1808

age 949, 955

age at births 167

AIDS 194

Akt 1711

alcohol 426

alcohol dehydrogenase 2039

alcohol drinking 182

allelic imbalance 2181

AlPcS_2a_ 2004

anal cancer 1221

anaplastic thyroid cancer 1899, 2216

androgen receptor 140

aneuploidy 1759

angiogenesis 89, 94, 1182, 1493, 1696, 1720, 1955

angiotensin 1247

anthracyclines 467, 475, 639

antiangiogenic 1599

antibiotics 594

antibody 1430

antihypertensives 1302

anti-idiotypic antibodies 1358

antineoplasic drugs 690

antisense 532

antivascular 1599

anxiety 990, 1393

apoptosis 120, 328, 359, 513, 681, 705, 736, 1089, 1188, 1247, 1430, 1467, 1593, 1773, 1955, 2185, 2225

Arg72Pro mutation 1144

array CGH 1553

arthroplasty 1298

asbestos 580

ascites and metastasis 1475

ascitic fluid 895

Ashkenazi Jewish melanoma families 2278

AT_1_ 1247

atomic force microscopy 1499

audit 55

autologous stem cell transplantation 1984

*β*1 integrin 1499

*β*3-integrin expression 41

*β*-catenin 1581

Barrett’s metaplasia 888

basic fibroblast growth factor 705

BAY 43-9006 1855

Bcl-2 family 449

benzo[*b*]thiophenesulphonamides 690

Bid 1459

Bim_EL_ 1773

bioconjugation 1442

biological marker 1231

biomarker 236, 1599

biotin 1261

birth interval 167

bisphosphonates 1869

bladder 125

bladder cancer 625, 645, 1276, 1906, 2145, 2153, 2262

bladder transitional cell carcinoma 80

BNP7787 1636

body composition 673

body shape 2042

bone marrow 503

bone pain 1869

bowel function 1209

*BRAF* 2032

brain metastases 815

BRCA in men 1288

*BRCA1* 857

breast 60, 613, 618, 949, 955

breast cancer 47, 55, 147, 167, 201, 225, 231, 342, 382, 467, 547, 553, 594, 597, 634, 906, 1013, 1283, 1293, 1366, 1524, 1531, 1614, 1621, 1720, 1869, 1922, 1948, 1955, 2039, 2049, 2102, 2206

breast cancer survival 961

breast carcinoma 120, 156, 631, 1261

breast neoplasms 2042

breast neoplasms/pathology 2201

breast tumours 389

breast volume 857

*BRG1* 1126

cachexia 1830

cadherin superfamily 2010

cadherin-16 2010

cancer 334, 1862, 2097, 2166, 2148

cancer cachexia 876

cancer genetics 1671

cancer incidence 201

cancer invasion 252

cancer mortality 580, 1329

cancer networks 1201

cancer prevention 971

cancer registries 188, 576, 1288

Cancer Research UK 1830

cancer risk 182, 572

cancer screening 265

cancer survival 1808

cancer therapy 1599

cancer vaccines 1358, 1421

capecitabine 246, 820, 2129

carboxylesterase 882

carboxypeptidase G_2_ 480

carcinogenesis 562, 888

carcinoma 15, 736

carcinoma *in situ* 1934

case–control studies, 594, 607, 1614, 1794, 2065, 2102

case-mix adjustment 55

CBS 838

CCL2 2024

CD1a 888

CD44 1767

CD4+ and CD8+ T-lymphocytes 651

CD4+ T-cell subsets 913

CD4+CD25+ T cells 913

CDC 342

CDK4 2278

CDKN2A/ARF 2278

cDNA array 1193

cDNA microarray 929

cell adhesion 366, 2010

cell culture 2166

cell cycle 7

cellular localisation 1702

cellular retinoic acid binding protein 696

central nervous system 747

centrosome aberrations 389

cervical cancer 41, 1800

cervical neoplasias 601, 1794

cervix carcinoma 449

cetuximab 1063

*CHEK2* 784

chemoembolisation 1862

chemoendocrine treatment 467

chemoprevention 513

chemoprophylaxis 867

chemoprotection 294

chemoradiation 284, 1372

chemoradiotheraphy 1341

chemoradiotherapy 655, 1221

chemotherapeutic agents 1430

chemotherapy 36, 294, 334, 350, 459, 475, 673, 799, 1372, 1644, 1650

chemotherapy response 147

childhood 2042

children 2084

chimeric antibody 1069

CHK2 784

cholecystectomy 1307

cholesterol 1785

chromatin 1165

chromosome 382

chronic B-cell lymphocytic leukaemia (B-CLL) 1517

chronic pain 225

chronopharmacology 1684

*cII* gene 873

circulating tumour cells 906

circulating tumour DNA 2181

cisplatin 246, 334, 475, 639, 827, 1084, 1149, 1352, 1611, 1636, 1684

c-kit 1398

classic Kaposi’s sarcoma 188

classification 847

clinical trial 15, 1358

coactivator 2286

co-expression 1906

cohort 176, 2070

cohort studies 182, 194, 580, 995, 1302, 1326, 1803, 2042

colon 736, 1078, 1310

colon cancer 722, 985, 1307, 1655, 1803, 1846, 2240

colorectal 60, 426

colorectal adenoma 1193

colorectal cancer 24, 430, 651, 832, 838, 1055, 1137, 1746, 1782, 1842, 2233

colorectal cancer and antiangiogenesis 350

colorectal metastases 729

colorectal neoplasms 259, 434

combination 1006

combination therapy 722

comet 1626

comfrey 873

communication 1671

comparative genomic hybridisation 935

complement 895

condom use 1388

conformal radiotherapy 488

corticosteroid 867

cost 60

Cox-2 2225

CPT-11 882, 1055

C-reactive protein 21, 625, 651

Crohn’s like reaction 1746

CTL 72

curative resection 21

cutaneous squamous cell carcinoma 1326

CXCR4 503

cyclin D1 1581

*Cyclin L1* 770

CYFRA 21 13

CYP26 696

cytokines 921, 1450

cytomegalovirus 747

cytotoxic potential 1702

cytotoxic T cells 1450

Danish cancer patients 995

Danish cancer register 995

DCIS 162, 389

DDS 1240

death from all causes 2089

delay 1959, 1971

dendritic cells 1182

denial 1393

depression 1393

deprivation 1279

desmosome 2153

detection rates 597

dexamethasone 1084

DGGE 1144

diagnosis 236, 1959, 1971

diet 971

dietary fibre 1803

diethylstilbestrol 1538

differential expression 2249

differentiation 751

discordant sites 1288

disease associations 1298

disparities 1842

distress 990

diuretics 1302

DLK1 1574

DNA damage 334, 1524, 1626

DNA damage response 784

DNA double-strand break repair 1089

DNA methylation 838, 1165

*DNMT3L* 1934

docetaxel 15, 645, 827, 2129

dose escalation 488

dose intensity 832, 1019

dose optimisation 1055

dosimetry 298

drug combinations 1063

drug development 1837

drug interaction 681

drug resistance 80, 334, 1084, 1149

ductal carcinoma *in situ* 1720

E6 290

E7 290

early invasive colorectal carcinoma 1193

Ebp1 140

E-cadherin 985, 2018

ECF 1650

ECOG-ps 1834

ecological studies 1279

ECTO-NOX 690

efficacy 1855

EGF receptor 366

EGFR 1069, 1737, 1846

EGFR mutation 1922

EGFRvIII 1069

elastase 89

ELF 2122

endocrine tumours 94

endometrial cancer 2059

endometrium 1098

endostatin 89, 729

endothelium 503, 1493

endothelin A receptor 2148

energy balance 673

England 194

Ep-CAM 342, 882, 1767

epidemiology 1276, 1302, 1326, 1782, 1785, 1803

epidermal growth factor 1690

epidermal growth factor receptor (EGFR) 334, 1063, 1110, 1341, 1877, 2266

*epidermal growth factor receptor(EGFR)* gene 1711

epigenetics 1165

epirubicin 1989

epothilone B 1006

EPR effect 1240

Epstein–Barr virus 1593

ER 147

ER *α* 2286

ER *β* 2286

ErbB receptors 140

ErbB2 1421

ERK1 2206

ERK2 2206

ethnic differences 1787

ethnicity 1393

etoposide 639, 1019, 1486

Ewing tumours 705

exosomes 305

expression 376

extra-nasal 1226

Eysenck personality questionnaire-revised 2089

EZH2 1754

familial breast cancer 784

familial thyroid carcinoma 1892

family 1321

fast recruitment 1679

fat 971

fatty acid 971

FELV 1650

fever therapy 421

FGFR3 320

FGFR4 320

fibroblast growth factor 2, 705

first-line chemotherapy 246

FISH 770, 1253

flat penile lesions 1388

fluorescence *in situ* hybridisation 382

FNAB 389

folate 838, 1524

followup 430

follow-up study 1276

food frequency questionnaire 971

fried foods 2065

fruit 1782, 2059

*γ*-irradiation 320

G-17 1581

galectin-1 1188

gamma-glutamyl transpeptidase 294

garlic 1773

gastric 2122

gastric cancer 1051, 1130, 1273, 1759, 1767, 2190

gastric carcinoma 1850

gastric MALT lymphoma 312

gastrin 1506

gastrointestinal tumour 1117

gefitinib 13, 1110, 1690, 1711, 1846

gelonin 2004

gemcitabine 445, 645, 815, 1084, 1352, 1684, 2139

gene delivery 1414

gene expression 1553, 1934, 2240

gene expression profiles 1130

gene expression profiling 1506, 1561, 1915

gene–environment interaction 2039

genetic 60

genetic susceptibility 967

genistein 80

genotype 838

geographic 1842

German population 1159

germline mutations 1144, 2278

germline variation 784

Glasgow osteosarcoma 1684

GLC_4_ 1459

glioblastoma 1247

glioma 747

glucocorticoids 876

GM-CSF 1019

Golestan 176

GPS 1834

grade 613, 625

Great Britain 587

Greece 396

*GRIM-19* 1892

groin nodes 222

growth factors 921, 1247, 1467, 1506, 1720, 1899

GRP 522

GTL2 1574

guidelines 867

H2AX 1089

haemoglobin beta (HBB) 2216

hair 1837

Hash-1 751

HB-EGF 1737

hDAB2IP 1117

head and neck cancers 539, 913, 1046, 1341, 1819

head and neck squamous cell carcinoma 770

heat shock protein 499

*Helicobacter pylori* 1273, 1759

*Helicobacter pylori* eradication 312

hepatitis-B virus 607, 935

hepatitis-C virus 607, 935

hepatocarcinogenesis 935

hepatocellular carcinoma 252, 628, 929, 935, 1690, 1754, 1825

hepatocyte growth factor 1506

HER-2 1261, 1421

HER-2/neu 72, 147, 1253

hereditary non-polyposis colorectal cancer (HNPCC) 1746

Hes-1 751

HGF 1078

HIF 94

high-dose 5-FU and leucovorin 1013

high-dose methotrexate 480

high-dose therapy 47

histology 847

histone acetylation 1117

histopathology 1366

HIV 194

HLA 1253

HLA-related genes 967

*hMLH1* 396

*hMSH2* 396, 2286

HNPCC 396, 2240, 2286

homeobox gene 2233

homocysteine 838

hormone replacement therapy 1293

hormone resistant 36

hormone therapy 2049

HOXB13 376, 2233

HPV 290

HPV type concordance 1388

H-Ras 80

hTERT 1942

hTR 1942

human papillomavirus (HPV) 265, 601, 891, 990, 1794, 1800, 2195

human prostate cancer 2018

Hürthle cell tumours 1892

hWAPL 290

hypericin 1406

hypoxia 94, 350, 655, 1696

IBDQ 1663

ifosfamide 1626

IGF-1 2097

*IGF1* genotype 857

IGF-1 levels 857

IGF-1 receptor 2097

IGFBP-3 1283

IGFBP-6 1538

IGF-I 1467

IGF-II 1283

IGF-II IGF-IR 1467

ILF 2122

image cytometry 389

imaging 1046

imatinib 1398

imatinib mesylate 1881

ImiDs 217

immune responses 1358

immunoglobulin heavy chain gene 312

immunohistochemistry 113, 278, 743, 747, 1231, 1253, 1720, 1767, 2201

immunostaining 942

immunosurveillance 895

immunotherapy 799, 843, 895, 1069, 1253, 1450

imprinting 1574

*in situ* breast cancer 162

*in situ* hybridisation 743, 747, 2024

*in vivo* microscopy 729

incidence 188

India 601

inducible gene expression 681

inflammation 1268, 1927

informed consent 1001

inherited predisposition 2278

inherited predisposition to breast cancer 1144

inhibitor of apoptosis 120

in-house cDNA microarray 1915

inoperable pulmonary malignancy 1825

insulin-like growth-factor-I (IGF-I) 1283

insulin-treated diabetes 2070

integration 2195

integrin 1475

integrin *β*1 102

intensity-modulated radiation therapy 1819

interaction 1006

interferon 1655

interleukin 921

interleukin-12 1450

intestinal injury 294

intracellular signalling 539

intratumoral infusion 1414

intravesical therapy 2145

invasion 102, 1955

invasive breast cancer 162

invasive cervical cancer 891

invasive ductal carcinoma 847

Iran 176

Iressa 1063

irinotecan 15, 259, 722, 820, 1055, 1846, 1850, 2122

irradiation 539

Italy 188, 572

Japanese 2089

Japanese women 2102

K562 1486

Kaposi’s sarcoma 572, 1467

Ki-67 147

kidney neoplasms 2266

Ki-ras 405

Korean 1273

KP372-1 1899

K-ras 1098

large phase III clinical trials 1679

large-core needle biopsy 231

larynx 2185

LCIS 162

LENT SOMA 1663

leucovorin 24

leukaemia 1298, 2084

levamisole 1655

LHRH receptor 366

limited resection 1033

linkage 194

lipolysis 876

lipopeptide 1421

lipoproteins 1406

liposomes 1421

liver cancer 572, 1307

liver metastases 1825

liver neoplasms 607

liver transplantation 1862

*LKB1* 1126

localised cancer 236

LOH 236

longitudinal 673

longitudinal study 1834

long-term 1800

loss of heterozygosity (LOH) 30, 1517

low-dose radiotherapy 1221

LPAAT-*β* 1729

luminescence 298

lung cancer 131, 580, 743, 942, 1033, 1084, 1089, 1231

lung neoplasms 1321

lymph nodes 156

lymphadenectomy 1051

lymphangiogenesis 1720

lymphatic metastasis/pathology 2201

lymphocyte infiltration 1746

lymphoma 1298, 1352, 1593

M30-Apoptosense™ Elisa 532

magnetic resonance 1599

malignancy 570, 867

malignant lymphoma 1349

malignant melanoma 201

mammaglobin 1948

mammographic screening 1366

mammography 156, 597, 949, 955, 961, 2102

management 459

MAPK pathway 2032

maspin 1130, 1948

mastectomy 55, 225

maternal hormone levels 1787

mathematical model 47

matrix metalloproteinases 252, 2171

MC-26 cells 1581

MCP-1 2024

MDM2 2097

meat 1310

medulloblastoma 359

melanoma 662, 1398, 1499, 2249

membrane vesicles 305

menstruation 1098

mesna 1636

mesothelioma 580, 587

messenger ribonucleic acid 259

MET 1078, 1906

meta-analysis 131, 430, 434, 935, 1033, 2049, 2076

metastases 1690

metastasis 449, 503

metastatic 673, 2122

metastatic breast cancer 475, 1989

metastatic disease 634

metastatic oesophageal cancer 2129

metastatic pancreatic cancer 2139

metastatic survival 1382

methotrexate 24

methotrexate toxicity 480

methylation 760, 942, 1117, 1486, 1574

methylation specific PCR 1117

mice 1684

microarray profiling gene expression pattern 80

microarrays 376, 618, 1149, 1545

micrometastases 222, 906

micrometastasis 1948

microsatellite instability (MSI) 1517, 1746, 2240

mismatch repair 1137

mitochondria 513, 1459

mitochondrial DNA 1268

mitochondrial proteins 1892

mitogen-activated protein kinases 2266

mitogenic signalling 921

mitosis 1773

mixture 2160

MLPA 396

*MO25α* 1126

molecular targeted agents 799

molecular therapy 1899

monoclonal antibodies 1442

mortality 241, 426, 587, 955

motility 1955

MS 838

MT201 342

MTHFR 838

mucinous adenocarcinoma 259

multiphoton laser scanning microscopy 729

mutation 30, 1711, 2032, 2160

MX2 1486

myeloma 217

myocardial infarction 1614

nasopharyngeal cancer 967

nasopharyngeal carcinoma 799, 1382

natural history 156

natural killer cell lymphoma 1226

natural resistance 1711

neoadjuvant chemoradiotherapy 1215

neoadjuvant chemotherapy 147

neoplasms 421, 570

neovascularisation 553

nested case–control study 1273

neural system tumours 2278

neuroblastoma 751, 1984

neuroendocrine tumours 1506

neuropilin-1 328

neurotoxicity 832

NF-*κ*B 711, 1137, 1593

NK105 1240

nonmelanocytic skin cancer 201

nonseminoma 1934

non-small-cell lung 15

non-small-cell lung cancer 13, 459, 1038, 1834

Notch 751

Nottingham Prognostic Index 847

novel treatments 217

NSAIDs 1137

NSCLC 775

NSCLC cells 681

nuclear and cytoplasmic staining 113

occupation 1276

Octreotide 1493

oesophageal cancer 176, 284

oesophageal neoplasms 2065

oestradiol 547

old age 1221

older women 1794

oleate 971

olfaction 1611

oligonucleotide array 2249

oligonucleotide microarray 1130

oncogenes 770, 2190

oncogenic viruses 743

oncology 421

ongoing mutation 312

oral chemotherapy 1989

oral neoplasms 2065

oral squamous cell carcinoma 30, 2181

oral vinorelbine 1989

organ metastasis 1746

osteoclast 1531

osteosarcoma 1358

ovarian 60

ovarian adenocarcinoma 2024

ovarian cancer 113, 271, 278, 305, 1026, 1149, 1475, 1729, 1737, 1927

ovarian neoplasm 895

ovarian surface epithelium 1927

overweight 2042

oxaliplatin 259, 655, 832, 1644

oxidative stress 1696

p21 131, 284

p53 30, 284, 1089, 1137, 2097

*P53* gene 434, 1144

p53 status 2114

p53R2 284

paclitaxel 320, 634, 639, 1240

paediatric 1626

palliative treatment 1038

pancreas 94, 1372

pancreatic cancer 21, 89, 405, 445, 921, 971, 1307, 2076

pancreatic carcinoma 2225

papillary 1545

papillary thyroid cancer 2216

Parkinson’s disease 201

parotid gland 539

patient 1959

patient motivations 1001

patients’ preferences 807

PDGF-R 1398

pegylated liposomal doxorubicin 628

PEI 1862

pelvic radiotheraphy 1663

peptide 328

perfusion 1406

peritoneal fluid 1737

permeability 1406

peroxisome proliferator-activated receptor 113

Peutz–Jeghers syndrome 1126

PgR 147

pharmacodynamic 1837

pharmacogenomics 259

pharmacokinetics 820, 1006, 1636, 1855, 2004

pharyngeal neoplasms 2065

phase I 820, 1636

phase I study 815, 2139

phase II 15, 445, 628

phase I/II cancer clinical trials 1001

phase III trial 488

phosphatidic acid 1729

phosphorylation 2206

photochemical internalisation 2004

photodynamic therapeutic agent 1702

photodynamic therapy 298, 1406, 1442, 2004

PI3-Akt 681

picropodophyllin 1467

plakoglobin 2153

plasminogen activation 1475

platinum pretreatment 1019

PNA 405

*Pneumocystis* pneumonia 867

polycomb group protein 1754

polymer micelles 1240

polymorphisms 30, 2039, 2262

pooled analysis 1288

population-based 182, 995

population-based study 1293

population-based survival 1808

positron emission tomography 655

post mastectomy pain 225

post-Chernobyl 1545

post-operative 1372

precounselling needs 1671

prediction 434, 587, 613

prediction response 618

predictive marker 2114

predictivity 1261

pregnancies 167

pre-hospital 1971

premenopausal 467

premenopausal breast cancer 857

prescriptions 594

primary care 1959

proband 1321

prognosis 271, 278, 434, 449, 576, 618, 847, 1026, 1231, 1906, 1955, 2181, 2206, 2225

prognostic factors 13, 131, 662, 1382, 1767

programmed cell death 120

progression 2018

proliferation 751, 1467, 1493, 1506, 1881

promoter hypermethylation 775, 2190

prospective cohort study 857, 2089

prospective study 1283, 1782

prostate 376, 426, 503, 2166

prostate cancer 36, 140, 320, 366, 488, 1159, 1538, 1819, 2171

prostrate cancer 499

proteasome inhibitors 217

proteasome proteolysis 711

protein biosynthesis 2266

protein degradation 711

protein tyrosine kinases 1906

proteolysis-inducing factor (PIF) 711

PSA 488

PTEN 1711

pTNM 847

public understanding 265

pyrrolizidine alkaloid 873

quality of life 668, 990, 1869

quantitative RT-PCR 532

quercetin 499

R116010 696

*Rab1a* gene 1915

race 2084

Rad51 1089

radiation 125

radiation therapy 1614

radiation toxicity 488

radical radiotherapy 41

radiofrequency 1825

radioresistance 2185

radiosensitisation 125, 815

radiotherapy 449, 459, 799, 1038, 1201, 1372, 2114

raltitrexed 445

Raman spectroscopy 2166

randomisation 807

randomised controlled trials 811

*RAS* 131, 2032

RAW 264.7 1531

reactive oxygen species 1759

real-time PCR 405, 547, 775, 2249

rearrangements 396

receptors 1720

receptor specificity 2148

record linkage 1298, 1307

rectal cancer 655, 1209, 1215, 2114

rectal carcinoma 1209

rectum 1310

recurrences 430, 1026

redox state 690

referral 1959, 1971

refractory 1352

regulatory T cells (T_reg_) 913

relapsed 1352

remnant stomach cancer 562

renal cell cancer incidence 1302

renal cell carcinoma 843, 1421, 1825, 2010

renal development 2010

renal transplant 572

replication errors positive phenotype (RER+) 1517

research 613

research spending 241

resection 1372

resveratrol 513

retinoic acid 696

risk 594, 1302, 1349

risk factors 1273, 2042, 2065

*RNASEL* 1159

RON 1906

RUNX3 562

rye 1803

S-1 2139

safety 1855

saline hydration 1636

salvage chemotherapy 1850

salvage surgery 430

salvage therapy 1019

sarcoma 2004

SCID mice 72

SCLC 522

screening 55, 156, 597, 949, 955, 1800

screening frequency 961

screening interval 961

second primary 570

secondary care 1959, 1971

secondary cytoreductive surgery 1026

second-line chemotherapy 15

segmentectomy 1033

selective chemotherapy 499

seminoma 1934

semiquantitative PCR 278

sentinel lymph node biopsy (SLNB) 662

sentinel lymph node biopsy/methods 2201

sequential hemi-body irradiation 1984

sequential hormonal therapy 1621

serine proteases 760, 2171

serological analysis of recombinant cDNA expression libraries 929

serous papillary uterine cancer 1561

serum biomarker 2018

serum DNA 2181

sex hormone 1877

sFGFR 320

shortening 668

short-term preoperative radiotherapy 1209

signalling 2153

signalling pathways 1690

singlet oxygen 298

siRNA 290

skeletal metastases 1869

skin 570

skin cancer 499

small-area geography 1279

small-cell lung carcinoma 1553

small-molecule therapy 1922

smear test 990

smell 1611

smoking 426, 1321

smoking tobacco 1326

smoky coal exposure 1321

SNAIL 252, 985

snuff dipping 1326

social deprivation 631

sociodemographic 1971

socioeconomic status 1279

solid tumours 1855

soluble vascular endothelial growth factor receptor-1 553

SOM230 1493

somatostatin receptors 1493

SPARC 942

SP-D 522

SP-G 522

spheroid 882

splicing variants 1942

spontaneous neoplasm regression 421

squamous cell 570

squamous cell carcinoma (HNSCC) 1046, 1341

SSCP 2024

SSI 389

ST14 278

stage 625

stage I non-seminoma 2107

Stat1 1149

statin 1593

statistical methods 576

stearate 971

stem cell 2160

stomach cancer 1307

stomach neoplasms 827

*STRADα* 1126

subsequent malignancy 1288

suicide 995

surgery 459, 1209

surgical decision-making 631

surrogate 955

survival 21, 131, 459, 625, 651, 1279, 1329, 1767, 1834, 2089

survival analysis 576

survival signals 705

survivin 212, 271, 359

Survivin isoforms 359

survivin-2B 212

survivin-DEx3 212

surviving 2225

susceptibility 784

systematic review 131, 434

T lymphocytes 305

TADG-15 278

tamoxifen 547, 1098, 1614

tandem base substitution 873

targeted agent 1855

targeted therapy 7

targeted treatment 217

taxonomy 613

TCF4 2233

telomerase 1881, 1942

testicular cancer 1934

testicular germ cell cancer 1787

testicular neoplasm 1785

Testisin 760

TFPI-2 775

thalidomide 217

therapeutic trials 1599

therapy 985

thermal ablation 1825

thioredoxin 350

thromboembolism 1349

thymidine phosphorylase 1696

thyroid 1545

thyroid cancer 1892

time since births 167

tissue microarray analysis 770

tissue microarrays 743

tongue squamous cell carcinoma 1915

topoisomerase 1486

toremifene 1098

toxicity 1663

TRAIL 736, 1430

TRAIL receptor 1 1430

transactivation 2286

transformation 522, 1078

transgenic rat 873

transitional cell carcinoma 645, 2262

translocation 382

trastuzumab 342, 1261

treatment 1226

trends 1201

trial 949

trial registration 811

TSA 751

tumorigenesis 212, 1268

tumoritropic behaviour 1406

tumour 421

tumour antigens 929

tumour lysates 72

tumour necrosis factor alpha 1690

tumour progression 366, 2249

tumour size 21

tumour suppressor 760

tumour suppressor genes 2190, 2216

tumour-associated NADH oxidase 690

tumour-immune escape 1188

turkmen 176

type 2195

type-II diabetes 2076

tyrosine kinase 1922

tyrosine kinase inhibitor 1877

tyrphostins 294

UFT 827

Uganda 1808

UK Europe 1329

ultrasound 231, 499

undescended testes 1787

undifferentiated thyroid cancer 1110

United Kingdom 1794

untreated phase III 1650

urban–rural differences 2084

urokinase plasminogen activator receptor 1475

uveal melanoma 2032

Vaizey 1663

validation 668

variation 55

vascular endothelial growth factor 553

vascular endothelial growth factor A 1182

vascular endothelial growth factor receptor-2 553

vascular invasion 1754

vasopressin 522

VDR 985

vegetables 1782, 2059

VEGF 328, 1467, 1531, 1696

VEGFR1 1531

VEGFR2 1531

vinorelbine 634

viral hepatitis 1268

viral load 891, 2195

virus dissemination 1414

vitamin D 985

volunteer studies 2148

VPA 751

vulval cancer 222

vulval squamous cell carcinoma 102

waiting times 1201

wedge resection 1033

weekly vinorelbine 1013

Western blot analysis 532

whole grains 1803

wild-type *K-ras* 1310

xenografts 722

Xeroderma Pigmentosum Group C (XPC) 2262

XIAP 532

XR5944 722

years of life lost 241

ZD1839 13, 125

ZD 4054 2148

zinc-*α*_2_-glycoprotein 876

